# A Novel Denoising Method for Mud Continuous-Wave Signals Based on Selective Ensemble Strategy with Particle Swarm Optimization

**DOI:** 10.3390/s25247594

**Published:** 2025-12-15

**Authors:** Chongjun Huang, Wenbo Cai, Dongxiao Pang, Yan Yang, Xingwang Du, Xi Duan, Yunxu Zhou

**Affiliations:** 1 Drilling & Production Technology Research Institute, CNPC Chuanqing Drilling Engineering Co., Ltd., Chengdu 610500, China; huangcj_ccde@cnpc.com.cn (C.H.); dongxx_zyy@cnpc.com.cn (D.P.); 2 School of Mechanical Engineering, Southwest Petroleum University, Chengdu 610500, China; 3 School of Sciences, Southwest Petroleum University, Chengdu 610500, China; yangyan0701@swpu.edu.cn (Y.Y.); 202411000126@stu.swpu.edu.cn (X.D.); duanxi_2001@swpu.edu.cn (X.D.); 200831010091@swpu.edu.cn (Y.Z.)

**Keywords:** measurement while drilling, mud continuous-wave signal, filtering processing, selective ensemble, particle swarm optimization

## Abstract

During drilling operations, mud continuous-wave signals suffer severe distortion at the surface receiver due to dynamic and complex background noise. Traditional noise cancelation methods face limitations in effectiveness and generalization. To address this, this paper proposes a particle swarm optimization (PSO)-based selective ensemble filtering strategy. It adaptively selects optimal filtering algorithms matched to the target scenario and assigns their weights, enabling complementary strengths from diverse filters. Preferred filtering methods are applied in parallel, generating initial signals as candidate solutions within the reconstructed ensemble. The PSO algorithm identifies the optimal matching weights for each particle and performs the optimal selective ensemble of these candidates. This process enhances generalization capability and achieves high-precision reconstruction of the continuous-wave signal in complex drilling environments. Experimental results demonstrate that the reconstructed signal using this method exhibits superior quality metrics and greater robustness compared to any single-filter output, validating the proposed approach’s effectiveness.

## 1. Introduction

Mud pulse telemetry (MPT) serves as one of the principal techniques for high-speed downhole data transmission [1]. Continuous mud pulse signals are widely employed due to their high-speed capability, reliable long-distance transmission, and cost-effectiveness [2]. However, as the transmission distance increases, the operational environment becomes more complex. Influenced by factors such as mechanical noise, drill string components, well depth, and mud properties, mud pulse signals are susceptible to varying degrees of distortion [3]. Consequently, measurement-while-drilling systems require advanced signal processing algorithms for effective data extraction and analysis [4,5,6,7].

To extract useful signals in complex noise environments, many researchers have leveraged the fact that downhole pulse signals and pump noise propagate in opposite directions. By computing the cross-correlation function of two signals simultaneously acquired from dual sensors, the time delay is determined, and the differential method has been successfully employed to separate pump noise from useful signals, thereby eliminating random noise interference between the two sensors [8,9,10,11]. However, the time delay is determined by the installation distance of the dual sensors and the pressure wave velocity in the drilling mud. Accurate determination of the pressure wave velocity is challenging and subject to fluctuations. Moreover, constraints such as cost and field installation limitations have hindered its widespread application in the drilling industry [12,13].

In response, Chen et al. [14] proposed a model-based pump noise suppression method that utilizes a single sensor and standard Kalman filter to eliminate pump noise, demonstrating excellent anti-interference performance. Additionally, in traditional single-sensor detection approaches, Wu et al. [15] employed a bandpass filter to remove out-of-band pump noise and other interference from continuous-wave signals, followed by the application of a Kalman filtering method to eliminate in-band pump noise and reconstruct the signal. Separately, Zhao [16] conducted an in-depth study on wavelet transform for signal denoising, which significantly improved signal processing efficiency.

In recent studies, the novel approaches that decompose input signals into intrinsic mode functions (IMFs) with inherent characteristics for noise elimination have gradually become a research focus in the field of continuous-wave mud pulse signal processing. Empirical mode decomposition (EMD) [17] and variational mode decomposition (VMD) [18] are the most representative methods. EMD adaptively decomposes a given input signal into several IMFs arranged from high to low frequency, each containing useful signals and noise within different frequency ranges. This enables combinations of modes to function as low-pass, high-pass, or band-pass filters, and the method has achieved remarkable success in engineering applications [19,20]. Based on EMD, Dragomiretskiy and Zosso invented VMD to overcome the issues of end effects and mode mixing. This method is grounded in a solid theoretical framework, constructing an objective function based on the assumption of minimizing the total bandwidth of the modes, and then extracting all modes and their corresponding center frequencies via optimization. Subsequently, numerous researchers have proposed various effective strategies to optimize the initial parameters of VMD, leading to significant improvements in processing results under complex noise interference [4,21].

In addition to filtering and decomposition paradigms, another important research direction in the field of signal enhancement revolves around signal estimation based on advanced optimization. Such methods formulate signal recovery as an inverse problem, typically incorporating sparsity constraints or complex prior models to achieve superior performance, and have demonstrated exceptional effectiveness in challenging scenarios such as compressed sensing [22].

Although various aforementioned single filtering techniques (e.g., Kalman filtering, wavelet transform, EMD/VMD and their variants) can achieve satisfactory denoising results under specific conditions and have become mainstream processing methods in the industry, they still face fundamental challenges in the dynamically complex environments of actual drilling operations. These methods inherently belong to the “single-model strategy” category, whose performance heavily relies on preset model parameters or decomposition orders. However, during cross-well sections or continuous drilling processes, drastic variations in factors such as weight on bit (WOB), rotational speed, formation properties, and drilling fluid performance lead to significant non-stationarity and time-varying characteristics of signal and noise. Consequently, any fixed-parameter filter or single-mechanism algorithm struggles to maintain optimal performance throughout the entire operation period; it may exhibit excellent performance at a certain stage but suffer a sharp degradation when unable to adapt to environmental changes at another stage. Due to differences in their underlying mechanisms, various denoising methods possess distinct capabilities in signal feature extraction and are suitable for different scenarios. Therefore, fully leveraging the complementary characteristics of multiple optimized algorithms in signal reconstruction, adaptively selecting filtering algorithms that match the target scenario, and effectively fusing the advantages of different algorithms represent a key breakthrough to overcome the bottlenecks of existing technologies.

Since its proposal, the Particle Swarm Optimization (PSO) algorithm has been widely proven to be a versatile and efficient meta-heuristic optimization method, demonstrating outstanding performance in solving complex optimization problems across multiple scientific and engineering fields, including next-generation communications [23], electromagnetics [24], resource allocation [25], and healthcare [26]. A common characteristic of these application scenarios is the need to find global optimal solutions within complex multi-parameter search spaces, which is highly consistent with the problem addressed in this paper. For the denoising task of mud continuous-wave signals, the core lies in adaptively selecting and weighting multiple heterogeneous filters, which essentially constitutes a complex parameter optimization problem. Building on PSO’s mature application experience in high-difficulty optimization scenarios, this paper proposes a PSO-based selective ensemble filtering strategy. We argue that the powerful global search capability of PSO demonstrated in fields such as 6G networks, electromagnetics, and medical logistics is equally applicable to dynamically finding the optimal fusion mode of filters, thereby providing a more robust solution for signal denoising in the highly dynamic and non-stationary environment of measurement-while-drilling (MWD) mud pulse telemetry.

In summary, to address the issues of poor denoising effectiveness and weak generalization ability of traditional noise elimination methods under complex working conditions such as cross-well sections or continuous drilling, a PSO-based selective ensemble filtering method is proposed. This method can dynamically and adaptively realize the optimal selection of filtering algorithms and their weight configuration matching the target scenario, thereby effectively fusing the advantages of different algorithms to achieve higher-quality signal denoising. The core innovations are as follows:

(1) For the first time, a selective ensemble strategy is proposed in the field of mud continuous-wave signal denoising, fully leveraging the complementarity of multiple excellent heterogeneous filtering algorithms in feature extraction and applicable scenarios.
(2) The PSO algorithm is introduced to dynamically and adaptively realize the selection of filtering algorithms and their weight matching, which are compatible with the target scenario. This enables our method to achieve data-driven and intelligent filter combination configuration based on real-time signal characteristics, breaking through the limitations of fixed-parameter filters or fixed ensemble strategies.

Compared with the standard single filtering techniques currently used in the industry, the proposed method does not rely on any fixed denoising model. Instead, through an adaptive fusion framework, it ensures that the system can automatically invoke and integrate the results of the best-performing filters when facing complex and variable noise environments, thereby achieving superior overall robustness, higher denoising accuracy, and stronger generalization ability. The remainder of this paper is structured as follows: Section 2 establishes models of continuous-wave signals and noise, and elaborates on the challenges in denoising such signals; Section 3 explains the principles and workflow of the proposed method; Section 4 presents experimental results on different signals, demonstrating the denoising effectiveness of the approach; and finally, the conclusion summarizes the study.

## 2. Establishing the Mud Continuous-Wave Signal Model and Analysis of Difficulty in Denoising

### 2.1. Establishing the Mud Continuous-Wave Signal Model

In a continuous-wave mud pulse telemetry system, a pulser installed near the drill bit converts downhole data into pressure fluctuations, which are then detected by pressure sensors mounted on the surface flowline [19]. During drilling operations, the reciprocating action of the mud pumps generates pump noise, which typically exhibits very high amplitude. Meanwhile, the system is subjected to mixed noise interference from downhole motors, bubbles in the drilling fluid, and moving drill string components. Consequently, the continuous-wave signal, already attenuated over the long transmission distance, exhibits a low signal-to-noise ratio (*SNR*).

Due to the reciprocating action of the mud pistons, pump noise tends to demonstrate a certain degree of periodicity [27]. The mud pump interference can be regarded as a superposition of multiple harmonic components and is expressed as follows [28]:

(1) $$p\left(t\right)=A_{0}+\sum_{k=1}^{M}A_{k}\sin\left(2\pi f_{k}t+\theta_{k}\right)$$

where $p\left(t\right)$ denotes the pump noise pressure signal at time *t*; $A_{0}$ represents the DC component or mean value of the pump outlet pressure, which represents the average level of the noise signal independent of harmonics. *M* is the highest harmonic order considered, $A_{k}$ is the amplitude of the *k*-th harmonic, $f_{k}$ is the frequency of the *k*-th harmonic, $2\pi f_{k}t$ constitutes the angular frequency of the *k*-th harmonic varying with time, and $\theta_{k}$ denotes the initial phase of the *k*-th harmonic.

The fundamental frequency of the pump noise is determined by the pump stroke rate, while the frequencies of its harmonic components are integer multiples of the pump stroke rate. The frequency of each harmonic of the pump noise interference can be expressed as follows [29]:

(2) $$f_{k}=\frac{k\cdot s}{60}$$

where $f_{k}$ is the *k*-th harmonic frequency, and *s* represents the number of rectangular pulses per minute output by the pump stroke sensor.

The mud channel noise $n\left(t\right)$ can be considered as a superposition of pump noise and random noise. Therefore, the continuous-wave signal collected on the ground can be modeled by the equation

(3) $$r\left(t\right)=s\left(t\right)+p\left(t\right)+\omega \left(t\right)$$

where $s\left(t\right)$ represents the ideal signal component and $\omega \left(t\right)$ denotes the random noise.

**Figure 1:**
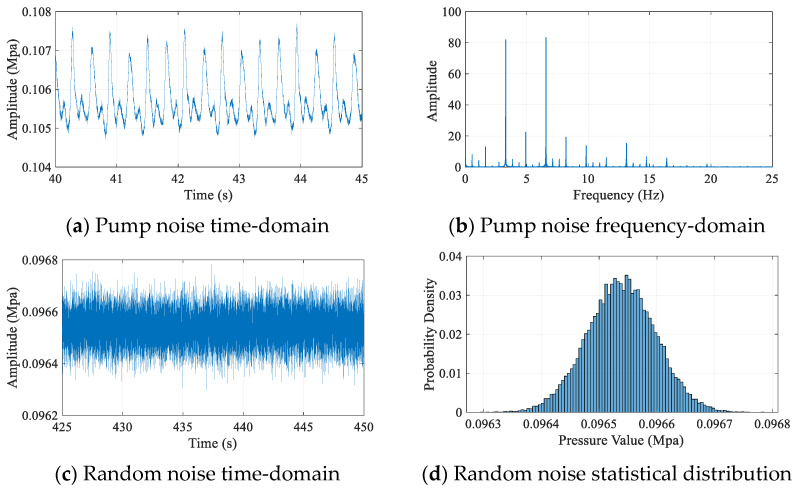
Characteristic spectrum of mud noise signal for the field measurements in actual wells.

For the field measurements in actual wells, the time-domain and frequency-domain characteristics of pump noise are shown in Figure 1a,b, respectively. It can be observed that the pump noise exhibits distinct periodicity, with its frequency-domain distribution demonstrating a comb-like structure. The time-domain waveform and statistical distribution of the random noise in the mud signal are presented in Figure 1c,d, indicating that the random noise can be reasonably approximated as following a Gaussian distribution. These observations verify that the aforementioned noise modeling formulation aligns well with practical conditions.

The comb-like spectral characteristics of pump noise shown in Figure 1b provide direct justification for employing band-pass filters in the denoising strategy. The fundamental frequency of the pump noise and its harmonics generate a series of narrowband interference peaks across the 1–20 Hz spectrum, with their energy particularly concentrated below 10 Hz. Since the frequencies of useful continuous-wave signals (e.g., 12 Hz and 18 Hz in our subsequent experiments) are typically designed to reside within the spectral gaps between these harmonic peaks, a well-designed band-pass filter whose passband covers the signal frequencies can effectively attenuate these strong harmonic components. This makes band-pass filtering a rational and essential technique, either as a standalone denoising step or as a critical component integrated with other methods (such as EMD or VMD) for initial noise suppression.

### 2.2. Analysis of Difficulty in Denoising

In practical engineering applications, the drilling process is inherently dynamic. This is manifested through variations in factors such as weight on bit, rotary speed, bit wear, wellbore trajectory adjustments, formation changes, drilling fluid properties, pump pressure fluctuations, and drill string vibrations. These factors cause significant temporal variations in signal characteristics like amplitude, frequency, phase, and *SNR*, resulting in strongly non-stationary and highly complex signal behavior. Consequently, no single noise model can accurately suppress noise interference across all scenarios, and it is challenging for any individual algorithm to maintain optimal performance under diverse complex noise backgrounds.

Different denoising methods exhibit varying capabilities in feature extraction and applicability due to their distinct mechanisms. For instance, Kalman filtering relies on an accurate model and is sensitive to model inaccuracies and changes in noise statistics [30]. Wavelet denoising depends on the proper selection of basis functions and thresholding rules, which may perform sub-optimally in handling abrupt changes in non-stationary signals [31]. Bandpass filters, such as FIR, Chebyshev, and Butterworth, require fixed passband settings and cannot adapt to noise with varying spectral characteristics [32]. Methods like EMD and VMD, which decompose signals into multiple intrinsic mode functions (IMFs) for noise removal, may lead to significant loss of useful signals and unsatisfactory denoising performance if parameters are not adjusted promptly according to the noise characteristics. Evidently, in the highly dynamic and complex environment of mud pulse signals, no single algorithm can consistently deliver optimal filtering performance across all time points and signal segments. An algorithm may perform excellently in one phase but poorly in another.

Inspired by the ensemble learning strategy proposed in the field of machine learning [33,34,35], which selects multiple high-performance classifiers as base classifiers and assigns weights for weighted voting integration, this study adapts the ensemble concept to enhance the effectiveness of continuous-wave signal processing in complex drilling environments. Compared to single-algorithm approaches, ensemble strategies theoretically reduce risk and improve robustness by leveraging the combined results of multiple algorithms.

However, a fixed-composition ensemble filtering method cannot adapt to the time-varying characteristics of signals and noise. Particularly during periods of drastic feature changes, such as pulse initiation/termination, sudden strong noise bursts, or abrupt channel condition variations, the outputs of certain algorithms may become highly unreliable. Fixed weighting schemes would incorporate these anomalous solutions into the fusion, potentially degrading overall performance to a level worse than that of a single algorithm in specific intervals. To address this limitation, this paper proposes a selective ensemble filtering strategy based on particle swarm optimization (PSO). This approach utilizes PSO to automatically adjust the algorithm combination scheme, enabling the ensemble filter output to maximally adapt to the target scenario’s performance requirements.

## 3. Materials and Methods

### 3.1. Particle Swarm Optimization (PSO)

PSO is a population-based optimization algorithm that seeks the optimal solution by simulating the collective behaviors of organisms such as bird flocks or fish schools [36]. In PSO, each potential solution is treated as a particle, and all particles navigate the search space with certain velocities. During the search process, both the position and velocity of each particle are continuously updated through iterative operations to ultimately converge toward the optimum. The velocity and position of each particle are updated using the following two equations:

(4) $$v_{id}^{k+1}=\omega v_{id}^{k}+c_{1}r_{1}\left(p_{id,pbest}^{k}-x_{id}^{k}\right)+c_{2}r_{2}\left(p_{d,gbest}^{k}-x_{id}^{k}\right)$$

(5) $$x_{id}^{k+1}=x_{id}^{k}+v_{id}^{k+1}$$

In the equations, $v_{id}^{k}$ represents the velocity vector of particle *i* in the *d*-th dimension at the *k*-th iteration; $x_{id}^{k}$ represents the position vector of particle *i* in the *d*-th dimension at the *k*-th iteration; $p_{id,pbest}^{k}$ represents the historical best position of particle *i* in the *d*-th dimension up to the *k*-th iteration; $p_{d,gbest}^{k}$ is the historical best position of the entire swarm in the *d*-th dimension up to the *k*-th iteration; *r*_1_ and *r*_2_ are random numbers within the interval [0, 1], introduced to enhance the randomness of the search process; *c*_1_ and *c*_2_ represent the individual learning factor and the social learning factor, respectively; and ω denotes the inertia weight.

The performance of each particle is evaluated through performance measurements specific to a particular problem, which is crucial for updating the particle’s position and velocity. Therefore, careful design of the fitness function is essential to effectively guide the improvement of particle performance.

### 3.2. PSO-Based Selective Ensemble Filtering Strategy

The proposed strategy constructs a set of candidate reconstructed signals for the current signal segment by processing it with each preferred filtering algorithm. Each solution represents a potential signal estimate derived from a distinct processing perspective. Subsequently, the PSO algorithm is employed to adaptively select and integrate the filtering algorithms that best match the target scenario. This approach maximizes the utilization of the advantages offered by different filtering methods, generating a reliable component closest to the ideal signal under the condition of minimized overall noise, thereby enhancing denoising accuracy and robustness.

A candidate algorithm library is constructed, comprising multiple filtering algorithms with strong complementary characteristics. These algorithms should be based on diverse principles (e.g., time-domain, frequency-domain, time–frequency-domain, linear, nonlinear) to cover the various possible signal morphologies and noise types. Specifically, the algorithm library adopted in this paper includes preferred filtering methods such as Kalman filtering, FIR filtering, Butterworth bandpass filtering, Chebyshev bandpass filtering, EMD processing, VMD processing, EMD combined with bandpass filtering (EMD–bandpass), and VMD combined with bandpass filtering (VMD–bandpass).

Subsequently, all algorithms in the library are used to process the continuous-wave pulse signal in parallel, obtaining the filtered signals *y_k_* (where k=1,2,…,N), which form the set $Y=\left\{y_{1},y_{2},\ldots ,y_{N}\right\}$ of candidate reconstructed signals for the current signal segment. Here, *N* represents the total number of preferred algorithms, and each filtering result corresponds to a potential signal estimate processed from a different perspective.

In the PSO algorithm, each particle acts as a selective weight allocator for the filtering results, carrying an *N*-dimensional position vector. The *i*-th particle can be represented as

(6) $${\vec{x}}_{i}=\left(x_{i,1},x_{i,2},\ldots ,x_{i,N}\right)$$

where each dimension $x_{i,k}$ corresponds to the candidate weight value for the *k*-th filtering algorithm. The position vector is further mapped into a probability distribution-based weight vector using the SoftMax function, denoted as $W_{i}=\left\{\omega_{i1},\omega_{i2},\ldots ,\omega_{iN}\right\}$, with the *k*-th element given by

(7) $$\omega_{ik}=\frac{e^{x_{i,k}}}{\sum_{j=1}^{N}e^{x_{i,j}}}$$

The weights obtained from the above Equation (7) satisfy $\sum_{k=1}^{N}\omega_{ik}=1$.

For this particle, the selective ensemble filtering output can be expressed as

(8) $$y_{i\_best}=\sum_{k=1}^{N}\omega_{ik}\cdot y_{k}$$

Finally, the optimal weight assignment for the different processing results is achieved by constructing a cost function between the particle and the filtering outputs. The cost function is designed as

(9) $$\begin{array}{ll} fitness\left({\vec{x}}_{i}\right) & =\frac{1}{N_{p}}\sum_{n=1}^{N_{p}}{\left[s\left(n\right)-y_{i\_best}\right]}^{2}\\ & =\frac{1}{N_{p}}\sum_{n=1}^{N_{p}}{\left[s\left(n\right)-\sum_{k=1}^{N}\omega_{k}\cdot y_{k}\right]}^{2} \end{array}$$

where *N_p_* denotes the signal length, and *s*(*n*) is the ideal signal. A smaller value of this function indicates a better weight allocation scheme for the particle.

Through the optimization process, the weight vector $W=\left\{\omega_{1},\omega_{2},\ldots ,\omega_{N}\right\}$ gradually converges to the optimal state. This weight vector inherently embeds a selection mechanism: weight $\omega_{i}$ close to 0 indicates that the solution of the *i*-th algorithm is effectively ignored or weakened in the current segment, while a larger value means its solution is prioritized and adopted, thereby forming a dynamic, data-driven selection mechanism.

**Figure 2:**
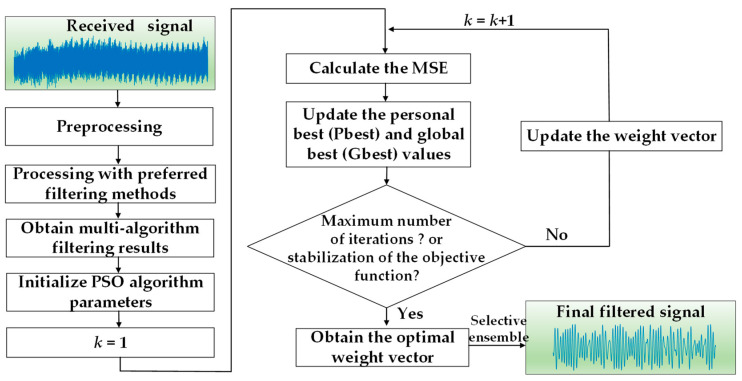
The overall workflow of the proposed PSO-based selective ensemble filtering algorithm.

The overall workflow of the proposed PSO-based selective ensemble filtering algorithm is illustrated in Figure 2. First, the received pressure signals of opposite pipes are subjected to preprocessing to remove DC components. Subsequently, the optimized filtering algorithms are adopted to process these signals sequentially, thereby forming a set of reconstructed signals. Next, parameters of the PSO algorithm are initialized, such as the number of iterations, lower and upper bounds of the search space, swarm size, dimensions, etc. The iteration process takes the cost function as the objective and continuously optimizes the candidate solutions. Such iterations are repeated until the maximum number of iterations is reached or the tolerance error is satisfied. Finally, selective ensemble filtering is performed using the obtained optimal weights to output the final filtered signal.

## 4. Experimental Result

In this section, the effectiveness of the proposed method is verified with both simulated and measured datasets. For the simulation processing part, to intuitively demonstrate the improvements in denoising performance of the proposed method, denoising experiments on mud continuous-wave signals were conducted under different noise conditions and different coding orders. Furthermore, measurement data from a certain oilfield in southwestern China is adopted to verify the effectiveness of the proposed approach.

### 4.1. Simulation Processing Example

In the experiment, the continuous-wave signals adopt BFSK encoding, with main frequencies of 12 Hz and 18 Hz and a transmission rate of 6 bit/s. Experiments were conducted on 30 groups of continuous-wave signal segments with different coding orders and noise conditions. The preferred base filtering algorithms mainly include 10 types, namely Gaussian filtering, EMD, Butterworth band-pass filtering, Chebyshev band-pass filtering, FIR filtering, wavelet processing, Kalman filtering, VMD, EMD + band-pass filtering, and VMD + band-pass filtering, which are sequentially labeled as filtering method 1 to filtering method 10.

**Figure 3:**
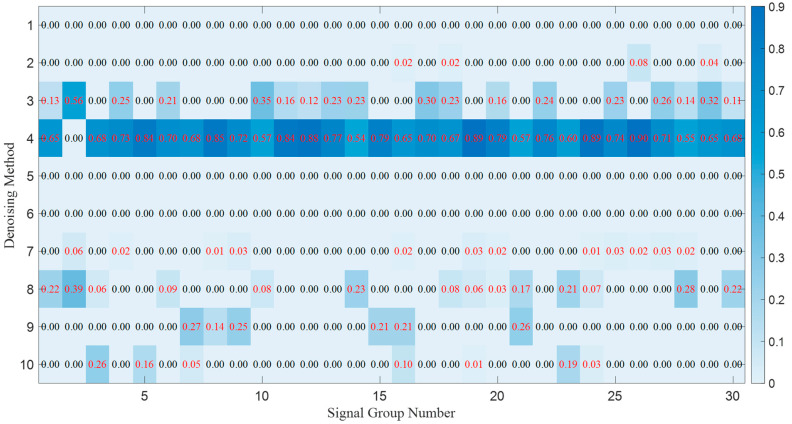
The optimal weights obtained from the PSO-based selective ensemble processing of 30 groups of noisy continuous-wave signals.

The initial parameter settings of the PSO algorithm are shown in Table 1. Among these parameters, the inertia weight is set to change dynamically, with an initial value of 0.9 that linearly decreases to 0.4, satisfying the following formula:

(10) ω=0.9−0.5×iter/iter_max

where iter denotes the number of iterations, and iter_max denotes the maximum number of iterations.

iter_max After performing parallel processing by different preferred base filtering algorithms, the optimal weights obtained from the PSO-based selective ensemble processing of 30 groups of noisy continuous-wave signals are illustrated in Figure 3.

The darker the blue background is, the larger the weight value is. The red values indicate the weights of the corresponding processing methods selected after PSO processing, and when the weight value is 0, it means that the processing result of this method is not selected. It can be observed that for different continuous-wave signals, the proposed algorithm can match the selective ensemble weights corresponding to the processing results of different base filters.

**Figure 4:**
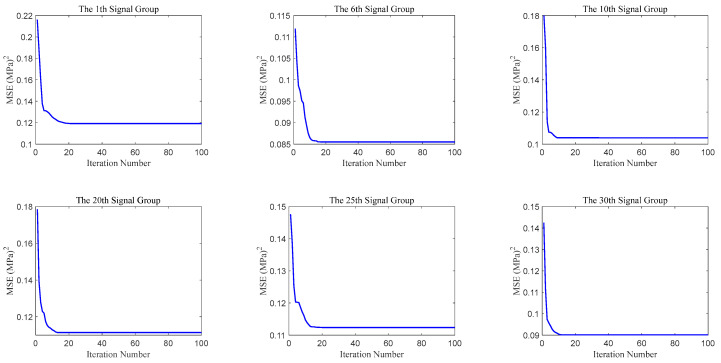
The variation in the cost function during the processing of 6 selected signal groups.

The variation in the cost function during the processing of six randomly selected groups of signals with iterations is illustrated in Figure 4. It can be observed that the cost functions continuously decrease with iterations and all reach a balance within the maximum number of iterations, indicating that the set maximum number of iterations meets the processing requirements.

To further quantitatively compare the quality differences of the reconstructed signals between the proposed algorithm and existing algorithms, the experiment employs Mean Square Error (*MSE*) and *SNR* to evaluate the filtering effects of different methods.

Let the ideal signal sequence be *s*(*n*), the denoised signal be $\hat{s}\left(n\right)$, and the signal length be *N_p_*; then the *MSE* is defined as

(11) $$MSE=\frac{1}{N_{p}}\sum_{n=1}^{N_{p}}{\left[s\left(n\right)-\hat{s}\left(n\right)\right]}^{2}$$

*SNR* refers to the ratio of the useful signal to the noise, specifically the ratio of the average power of the useful pulse signal to the average power of the system noise. The larger the *SNR* value, the higher the proportion of the useful pulse signal, and accordingly, the better the quality of the denoised signal and the more superior the denoising effect. The formula for *SNR* is as follows:

(12) $$SNR=10\times \mathrm{lg}\left(\frac{P_{s}}{P_{n}}\right)$$

where $P_{s}$ denotes the power of the signal, and $P_{n}$ denotes the power of the noise.

**Figure 5:**
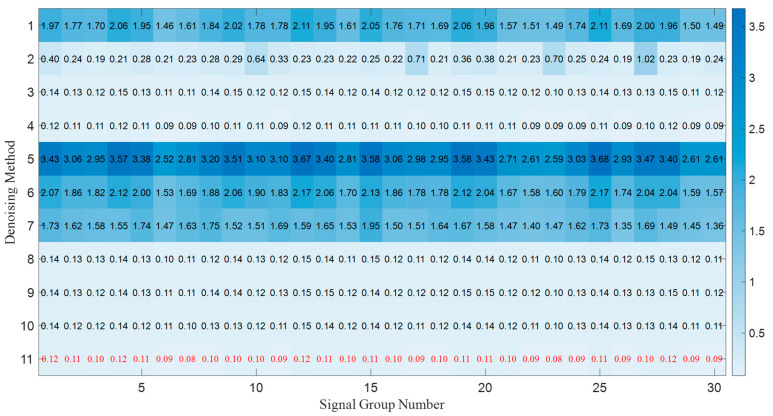
The *MSE* of 30 groups of signals after filtering via different methods.

**Figure 6:**
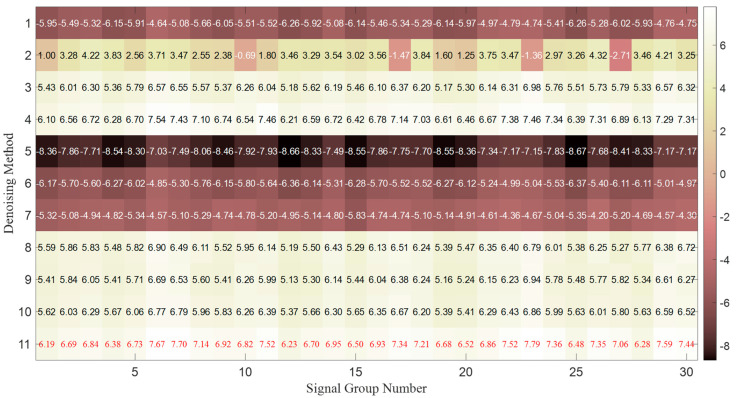
The *SNR* of 30 groups of signals after filtering via different methods.

The *MSE* and *SNR* results of the reconstructed signals from 30 groups of continuous-wave signals processed by different methods are illustrated in Figure 5 and Figure 6. Filtering methods 1 to 10 correspond to the 10 base filtering methods predefined at the beginning of the experiment. Filtering method 11 refers to the selective ensemble filtering method based on the optimal weights determined in Figure 3, i.e., the method proposed in this paper.

It can be observed that, compared with any individual base filter, the proposed method in this paper achieves the smallest *MSE* and the largest *SNR* for the reconstructed signals after processing 30 groups of signals under different coding orders and noise conditions, exhibiting excellent denoising performance and generalization ability.

**Figure 7:**
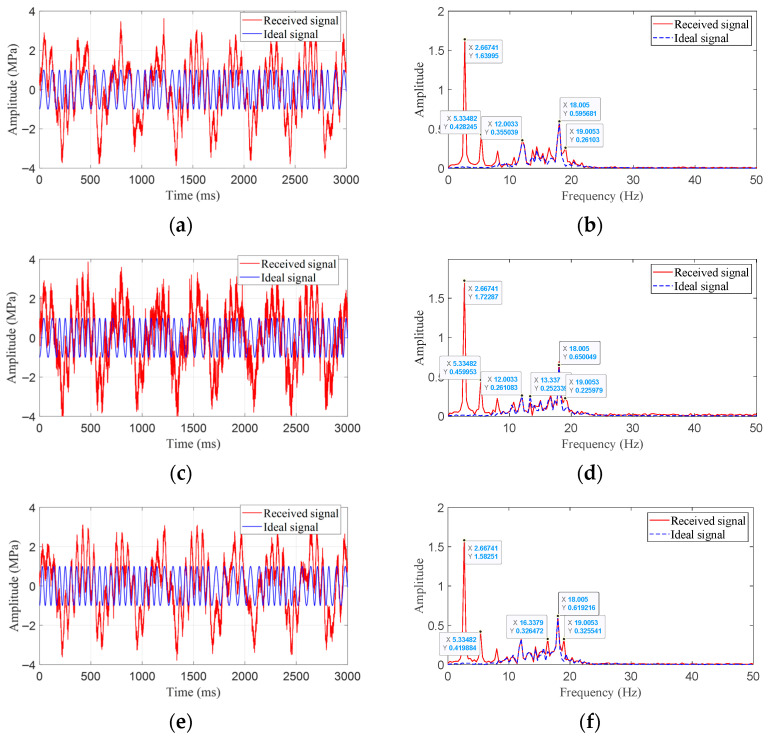
The time-domain and frequency-domain diagrams of the randomly selected signals and their corresponding ideal signals. (**a**) Time-domain diagram of the 1st randomly selected signal group. (**b**) Frequency-domain diagram of the 1st randomly selected signal group. (**c**) Time-domain diagram of the 2nd randomly selected signal group. (**d**) Frequency-domain diagram of the 2nd randomly selected signal group. (**e**) Time-domain diagram of the 3rd randomly selected signal group. (**f**) Frequency-domain diagram of the 3rd randomly selected signal group.

To further visualize the processing effects, three groups of signals are randomly selected for filtering effect analysis. The visualization results of the three received signals and their corresponding ideal signals in the time domain and frequency domain are shown in Figure 7.

As can be seen from Figure 7, these groups of signals are all submerged in noise. Without filtering processing, it would be difficult to recover the downhole encoded data.

After processing by the PSO-based selective ensemble algorithm, the weight vectors of the three randomly selected groups of signals are as follows:

(13) $$\left\{\begin{array}{l} \vec{W_{1}}=\left\{0,0,0,0.6765,0,0,0,0,0.2720,0.0515\right\}\\ \vec{W_{2}}=\left\{0,0,0.3529,0.5720,0,0,0,0.0751,0,0\right\}\\ \vec{W_{3}}=\left\{0,0.0357,0.3192,0.6452,0,0,0,0,0,0\right\} \end{array}\right.$$

Specifically, the first group of signals automatically matched the filtering results of three methods, namely Chebyshev band-pass filtering, EMD + band-pass filtering, and VMD + band-pass filtering, and we performed selective ensemble processing with different weights. The second group of signals automatically matched the filtering results of Butterworth band-pass filtering, Chebyshev band-pass filtering, and VMD, followed by selective ensemble processing with different weights. The third group of signals automatically matched the filtering results of EMD filtering, Butterworth band-pass filtering, and Chebyshev band-pass filtering, and we conducted selective ensemble processing with different weights.

The quality indicators of signals processed by different methods are shown in Table 2. Quantitative evaluation results indicate that after processing by the PSO-based selective ensemble algorithm, the reconstructed signals achieve the lowest *MSE* and the highest *SNR*, with optimal quality indicators, thus exhibiting excellent robustness.

To explicitly illustrate the performance improvement achieved by the selective ensemble method over the best single base filter, we performed an additional analysis on all 30 signal groups. Table 3 compares the results of the best-performing individual base filter against our proposed PSO-based selective ensemble method for three randomly selected signal groups. The results show that, across all tested signal groups, the selective ensemble method consistently achieves simultaneous improvements in both *MSE* and *SNR*. Specifically, for the 1st group, *MSE* was reduced by 6.09% and *SNR* increased by 3.70%; for the 2nd group, *MSE* decreased by 6.31% and *SNR* improved by 4.32%; and for the 3rd group, *MSE* was lowered by 6.75% while *SNR* rose by 1.84%. These results convincingly demonstrate that the selective ensemble strategy effectively leverages the complementary strengths of different base filters, yielding superior denoising performance compared to any individual base filter.

**Figure 8:**
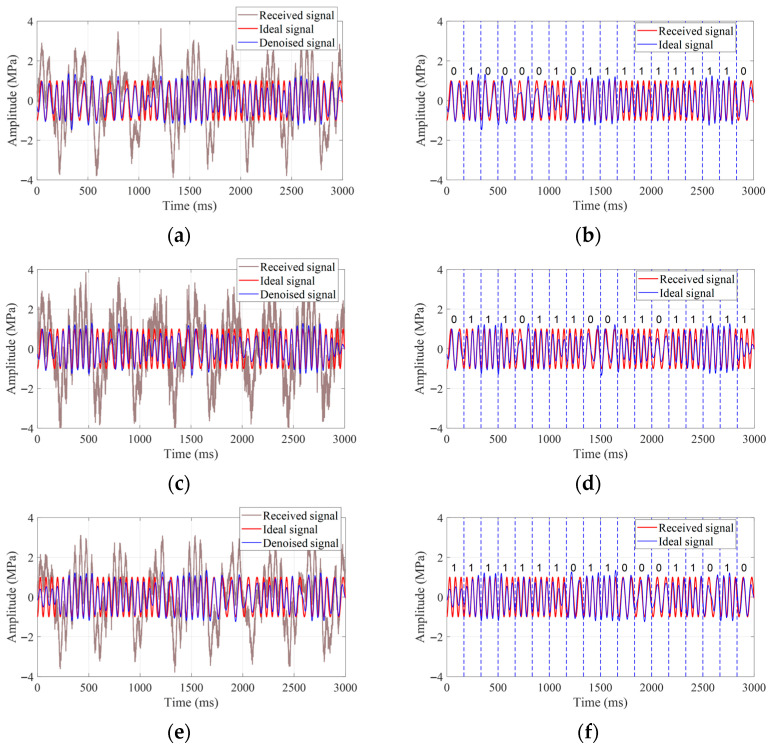
The final denoising effect and decoding results of the randomly selected signals. (**a**) The final denoising effect of the 1st randomly selected signal group. (**b**) The final decoding result of the 1st randomly selected signal group. (**c**) The final denoising effect of the 2nd randomly selected signal group. (**d**) The final decoding result of the 2nd randomly selected signal group. (**e**) The final denoising effect of the 3rd randomly selected signal group. (**f**) The final decoding result of the 3rd randomly selected signal group.

The visualizations of the final filtering results and the decoding results are shown in Figure 8.

It is evident that the noise-submerged continuous-wave signals, after being processed by the proposed method, yield reconstructed signals with optimal quality indicators, exhibiting the closest characteristics to the ideal signals. Meanwhile, the decoding accuracy of all three groups of signals reaches 100%, showing a high degree of consistency with the original codes in terms of decoding results.

The 30 groups of signals used in the experiment all have a transmission rate of 6 bit/s, with each group lasting 3 s, totaling 540 symbols. Statistical analysis of the processing results of all signals reveals that there are decoding errors in two symbols, consequently achieving an overall decoding accuracy of 99.6%.

### 4.2. Real Data Processing Example

**Figure 9:**
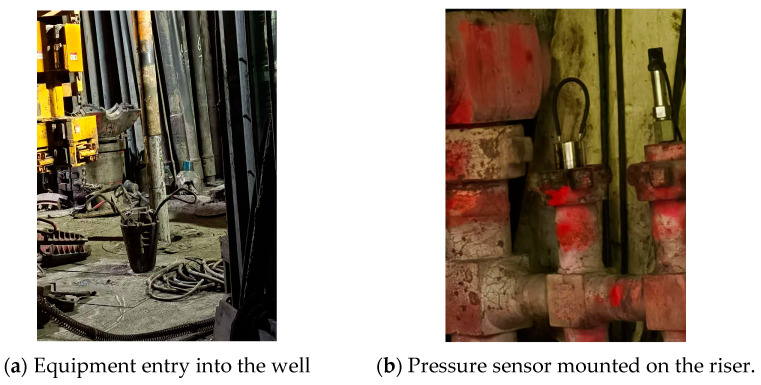
Field diagram of the data acquisition wellsite.

The experimental data were acquired from a wellsite in Southwest China, employing BFSK encoding with central frequencies of 12 Hz and 18 Hz, and a transmission rate of 4 bit/s. Figure 9 shows the field diagram.

**Figure 10:**
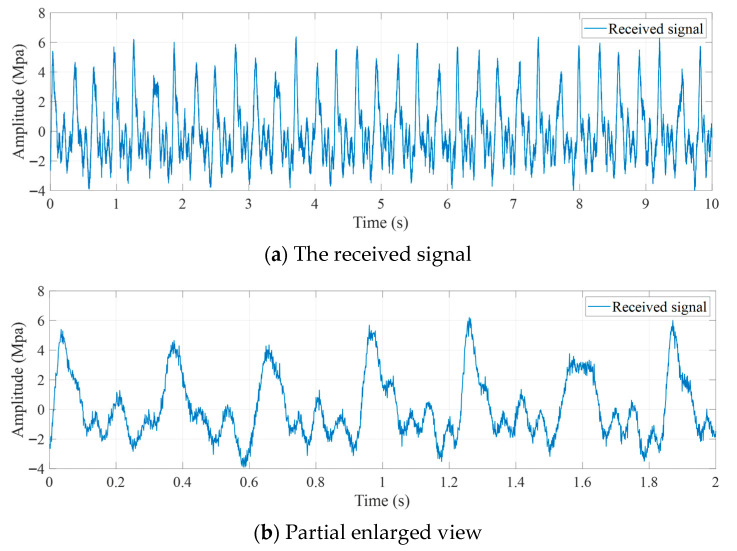
The received signal collected by a pressure sensor mounted on a riser.

After elimination of the direct current component, the pressure sensor, with a sampling rate of 1000 Hz, received the mud continuous-wave signal shown in Figure 10.

Consistent with prior experimental procedures, the preferred base filtering algorithms, namely Gaussian filtering, EMD, Butterworth band-pass filtering, Chebyshev band-pass filtering, FIR filtering, wavelet processing, Kalman filtering, VMD, EMD + band-pass filtering, and VMD + band-pass filtering, are sequentially labeled as filtering method 1 to filtering method 10. And the initial parameter settings of the PSO algorithm are shown in Table 1.

After processing with the PSO-based selective ensemble algorithm, the optimal weights matched according to the characteristics of the measured signal are determined as

(14) $$\vec{W}=\left\{0,0,0,0,0,0,0.0449,0.2412,0,0.7139\right\}$$

This indicates that the signal automatically matched the filtering results of three methods—Kalman filtering, VMD, and VMD + band-pass filtering—and we performed selective ensemble processing according to the weights.

Based on the known encoding sequence, the ideal signal of this mud continuous-wave signal is reconstructed, and *MSE* and *SNR* are employed to quantitatively evaluate the final filtering effect. The quality indicators of the received signal processed by different methods are shown in Table 4. Quantitative evaluation results indicate that after processing by the PSO-based selective ensemble algorithm, the reconstructed signal achieves the lowest *MSE* and the highest *SNR*, demonstrating superior quality indicators compared to all preferred base filtering methods.

**Figure 11:**
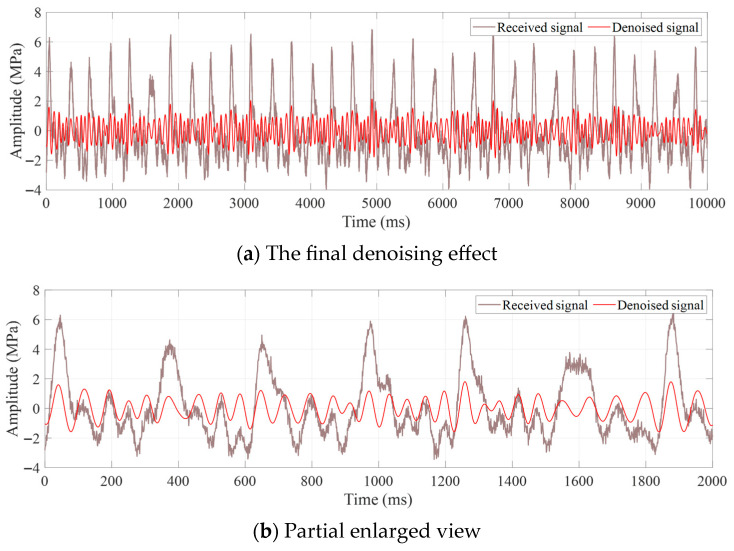

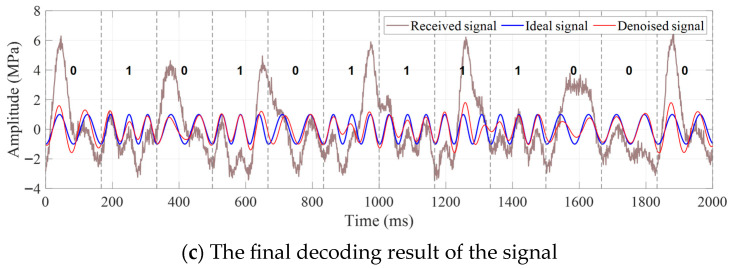
The final denoising effect and decoding result of the received signal.

The final denoising effect and corresponding partial decoding results of the signal are illustrated in Figure 11. It can be observed that the filtering results of the received signal overwhelmed by noise have the highest degree of consistency with the ideal signal, and its decoding results are consistent with the encoding, verifying the effectiveness of the proposed method.

### 4.3. Analysis of Algorithm Runtime and Practicality

#### 4.3.1. Theoretical Analysis of Computational Complexity

The time complexity of the proposed algorithm primarily depends on the signal length *L*, population size *S*, maximum number of iterations *T*, dimensionality *D* of the optimization problem, and the computational cost of the fitness function evaluation. In this work, D equals the number of base filters, which is a constant value of 10. During each iteration for each particle, the dominant computational cost arises from evaluating the fitness function (i.e., the cost function defined in Equation (9)). This evaluation involves the following steps:

(1) Using Equation (8), the *N* candidate signals are fused by weighting them with the current weight vector $W_{i}=\left\{\omega_{i1},\omega_{i2},\ldots ,\omega_{iN}\right\}$, which has a computational complexity of $O\left(N\times L\right)$.
(2) Compute the mean squared error between the fused signal and the ideal reference signal, which has a computational complexity of $O\left(L\right)$.

Therefore, the computational complexity of a single fitness function evaluation is $O\left(N\times L\right)$. Since *N* is a constant, this simplifies to $O\left(L\right)$. Consequently, the overall time complexity of the PSO algorithm is the product of the population size, the number of iterations, and the cost per fitness evaluation, i.e., $O\left(S\times T\times L\right)$. This indicates that the algorithm’s runtime scales linearly with the signal length, demonstrating favorable predictability and controllability.

#### 4.3.2. Runtime Statistics

All experiments were conducted on a personal computer equipped with an Intel Core i5 processor (clock speed 2.40–2.42 GHz) and 16 GB of RAM, implemented in MATLAB R2023a. We tested the algorithm on 30 signal segments, each lasting 3 s with a sampling rate of 1000 Hz (i.e., *L* = 3000). The population size was set to *S* =4 0, and the maximum number of iterations was *T* = 100. Experimental results show that the average processing time for a single signal segment using the PSO-based selective ensemble method was 0.2635 s, which aligns well with the theoretically predicted linear complexity and confirms the algorithm’s capability for near-real-time processing.

#### 4.3.3. Discussion on Online Practicality

Prototype system tests conducted on a standard personal computer demonstrate that the algorithm processes a 3 s signal segment in approximately 0.2635 s on average. This performance is sufficient to meet the requirements of applications that are not highly sensitive to latency—such as near-real-time monitoring of drilling parameters, high-precision post-processing analysis, and system validation. To better align with real-world drilling scenarios, a periodic weight-update strategy can be adopted: for example, updating the ensemble weights once every ten minutes and reusing these weights for selective ensemble fusion during the intervening period. Given that decision-making cycles in drilling operations typically occur on the order of minutes, this strategy already holds significant engineering value.

In summary, the selective ensemble approach exhibits well-defined computational characteristics and achieves runtime efficiency that satisfies practical engineering demands, demonstrating strong potential for real-world deployment. Integrating this method into MWD systems could enable online, highly robust signal processing capabilities.

## 5. Discussion and Conclusions

A PSO-based selective ensemble denoising method for mud continuous-wave signals is proposed to address the poor adaptability of single, fixed-parameter filters under highly dynamic drilling conditions. By processing each signal segment with multiple preferred filtering algorithms, constructing a set of candidate reconstructed signals, and then using PSO to adaptively select and weight these candidates, the method fully exploits the complementary advantages of different filters and generates a fused signal that is closest to the ideal one under minimized overall noise.

Experimental results show that the reconstructed signal consistently achieves the smallest *MSE* and largest *SNR* compared with any individual base filter. The decoding accuracy reaches 99.6% for 540 symbols in 30 simulated experiments and 100% for measured data, even when the signal is severely submerged in noise, demonstrating excellent denoising performance, robustness, and engineering feasibility.

Compared with traditional fixed filtering strategies, the proposed method breaks through the applicability limitations of single algorithms and significantly improves generalization in complex noise scenarios. Nonetheless, its performance remains dependent on the quality of the base filters. Future work will focus on developing and integrating more advanced base filtering algorithms to further enhance denoising performance and better cope with complex drilling environments, as well as to extend the applicability of the method to broader signal processing tasks.

## Figures and Tables

**Table 1:** The initial parameter settings of the PSO algorithm.

Parameter	Value	Parameter	Value
swarm size	40	individual learning factor *c*_1_	1.5
dimensions	10	social learning factor *c*_2_	1.5
inertia weight	0.9→0.4	iter_max	100

**Table 2:** The quality indicators of signals processed by different methods.

Number	Denoising Method	The 1st Signal Group		The 2nd Signal Group		The 3rd Signal Group	
		*MSE*	*SNR*	*MSE*	*SNR*	*MSE*	*SNR*
1	Gaussian filter	1.6108	−5.0805	1.7785	−5.5102	1.4972	−4.7505
2	EMD	0.2273	3.4708	0.6407	−0.6948	0.1899	3.2459
3	Butterworth	0.1107	6.5486	0.1184	6.2553	0.1101	6.3204
4	Chebyshev	0.0904	7.4264	0.1109	6.5410	0.0933	7.3092
5	FIR	2.8070	−7.4926	3.0991	−7.9221	2.6068	−7.1686
6	Wavelet processing	1.6926	−5.2956	1.9001	−5.7976	1.5859	−4.9689
7	Kalman filter	1.6289	−5.0991	1.5121	−4.7783	1.4531	−4.3006
8	VMD	0.1123	6.4864	0.1272	5.9461	0.1151	6.7189
9	EMD + band-pass	0.1113	6.5254	0.1184	6.2567	0.1091	6.2745
10	VMD + band-pass	0.1047	6.7903	0.1182	6.2641	0.1095	6.5227
11	PSO-based selective ensemble	0.0849	7.7014	0.1039	6.8237	0.0870	7.4403

**Table 3:** Comparison results between the optimal single filter and the selective ensemble method.

Signal Group	Optimal Single Base Filter		Elective Ensemble Method		Improvement *MSE*	Improvement *SNR*
	*MSE*	*SNR*	*MSE*	*SNR*		
1st Group	0.0904	7.4264	0.0849	7.7014	6.09%	3.70%
2nd Group	0.1109	6.5410	0.1039	6.8237	6.31%	4.32%
3rd Group	0.0933	7.3092	0.0870	7.4403	6.75%	1.84%

**Table 4:** The quality indicators of the signal processed by different methods.

Number	Filtering Method	*MSE*	*SNR*
1	Gaussian filter	3.6335	−8.6052
2	EMD	1.5116	−4.7749
3	Butterworth	0.1557	5.0745
4	Chebyshev	0.1890	4.2339
5	FIR	5.6002	−10.4839
6	Wavelet processing	3.8655	−8.8739
7	Kalman filter	2.5762	−7.0410
8	VMD	0.1701	4.6909
9	EMD+ band-pass	0.1537	5.1310
10	VMD+ band-pass	0.1448	5.3917
11	PSO-based selective ensemble	0.1371	5.6308

## Data Availability

The original contributions presented in this study are included in the article. Further inquiries can be directed to the corresponding author.
